# Prognostic power of global 2D strain according to left ventricular ejection fraction in patients with ST elevation myocardial infarction

**DOI:** 10.1371/journal.pone.0174160

**Published:** 2017-03-23

**Authors:** Myung-Jin Cha, Hyun-Sook Kim, Seong Hwan Kim, Jae-Hyeong Park, Goo-Yeong Cho

**Affiliations:** 1 Division of Cardiology, Department of Internal Medicine, Cardiovascular Center, Seoul National University College of Medicine, Seoul National University Hospital, Seoul, South Korea; 2 Cardiovascular Division, Department of Internal Medicine, Hallym University Medical Center, Anyang, South Korea; 3 Division of Cardiology, Department of Internal Medicine, Korea University Medical Center, Anam, South Korea; 4 Cardiovascular Division, Department of Internal Medicine, Chungnam National University Hospital, Daejeon, South Korea; 5 Division of Cardiology, Cardiovascular Center, Seoul National University Bundang Hospital, Seongnam, South Korea; Scuola Superiore Sant'Anna, ITALY

## Abstract

**Backgrounds:**

We aimed to evaluate the predictive power of longitudinal and circumferential fibers according to left ventricular ejection fraction (LVEF) in successfully reperfused acute ST elevation myocardial infarction (STEMI) patients.

**Methods:**

Total 691 patients (age 59±13, 20% female) underwent clinical evaluation and conventional and strain echocardiography (Global longitudinal strain (GLS), global circumferential strain (GCS)). The clinical outcome was defined as the composite of death, hospitalization for heart failure, non-fatal myocardial infarction, and ventricular arrhythmia.

**Results:**

During a follow-up of 39±19 months, there were 47 (6.8%) clinical events. In multivariate Cox models adjusted clinical risk factors, age (HR 1.08, p = 0.001) and GLS (HR 1.37, p = 0.001) were independent predictors. The addition of GLS resulted in significant incremental improvement in the predictive value on LVEF (χ^2^ = 31.8→45.8, p<0.001), although GCS offers no additional benefit. In the subgroup analysis according to LVEF, adjusted with clinical factors, GLS was significant predictive for outcome for the patients with mildly depressed (LVEF 40–50%, HR 2.25, p<0.001) and significantly depressed (LVEF<40%, HR 1.28, p = 0.016) systolic function, although GCS and LVEF lost their power with LVEF<40%. For the patients with preserved LVEF (>50%), GLS, GCS and LVEF did not show significant predictive power.

**Conclusions:**

GLS is a most powerful predictor of outcome in successfully reperfused STEMI patients, especially with depressed LV dysfunction, although GCS and LVEF lost their predictive power for the patients with significantly depressed LV function. However, GLS did not predict outcome for the patients with preserved LVEF (>50%).

## Introduction

Left ventricular (LV) systolic function is an important prognostic marker after ST elevation myocardial infarction (STEMI).[1] Left ventricular ejection fraction (LVEF) based on ventricular systolic and diastolic volume change is the traditional measuring method of systolic function. However, echocardiographic assessment of LVEF is subjective, especially when the endocardial border cannot be clearly defined, and there are reports concerning technical limitations of LVEF for clinical risk prediction in STEMI patients. [2, 3]

LV systolic function is a complex, coordinated action involving longitudinal contraction, circumferential shortening, and radial thickening. Two-dimensional (2D) strain based on speckle tracking is an emerging innovative method providing information about the functional status of the left ventricle after AMI. Global longitudinal strain (GLS) has been discussed in numerous studies as a superior marker compared with LVEF for cardiac events,[4] functional recovery or irreversible remodeling after acute myocardial infarction (AMI).[5, 6] Moreover, even though the echocardiographic estimation of LVEF is commonly trusted, few accuracy data are available for subjects with depressed LV systolic function.[7]

Contraction of muscle fibers in the mid-wall, which is linearly related to circumferential strain,[8] may better reflect intrinsic contractility than contraction of fibers in the endocardium.[9] In heart failure study by Cho et al, global circumferential strain (GCS) showed better prognostic power than longitudinal strain.[10] However, there are few data on the use of circumferential strain as a predictor in AMI patients.

In contrast, GLS has been introduced as a prognostic marker in AMI patients with preserved LV systolic function. However, the study had an inclusion criteria of EF > 40%, therefore including patients with actual LV dysfunction.[4, 11] In the current study, we divided LV systolic function into preserved (EF > 50%), mild decreased (40–50%), and significantly decreased (EF < 40%) groups to verify the prognostic power of GLS and GCS.

We hypothesized that both GLS and GCS will play an important role predicting clinical events in STEMI patients regardless of LV systolic dysfunction. We sought to evaluate the predictive power of longitudinal and circumferential fibers according to LV systolic function in successfully reperfused STEMI patients with low clinical risk.

## Methods

### Patient population

**Fig 1:**
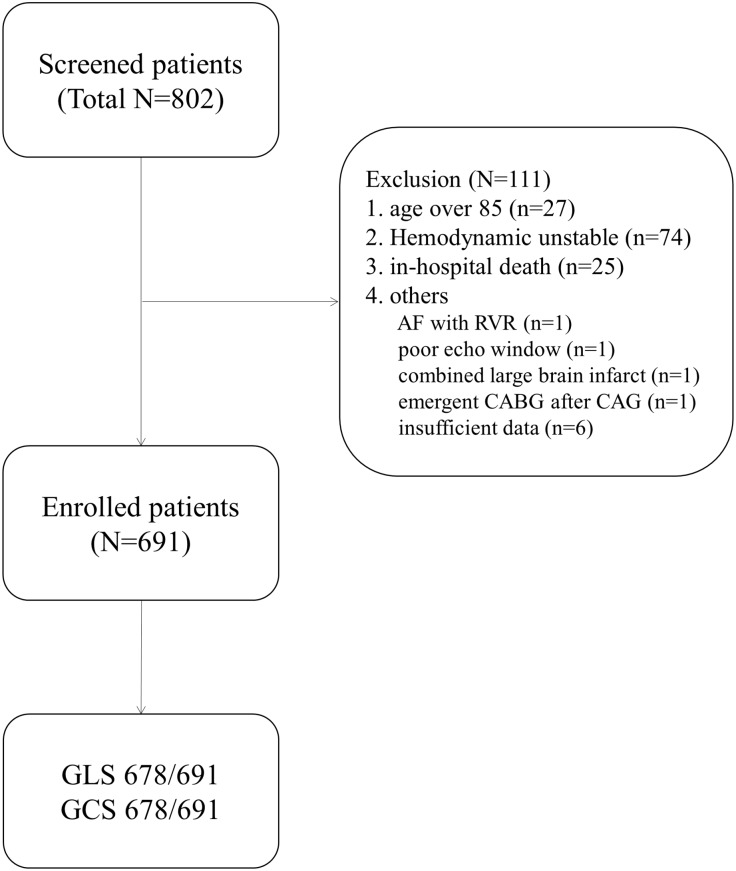
Patients flow with exclusion criteria. IABP, intra-aortic balloon pump; ECMO, Extracorporeal membrane oxygenation; CRRT, continuous renal replacement therapy; AF, atrial fibrillation; RVR, rapid ventricular response; CABG, Coronary Artery Bypass Graft surgery; CAG, coronary arteriography.

Between April 2006 and July 2012, 802 patients admitted for STEMI were enrolled at four cardiovascular centers. STEMI was defined following previous clinical guidelines.[12] STEMI is a clinical syndrome defined by characteristic symptoms of myocardial ischemia in association with persistent electrocardiographic ST-segment elevation and subsequent release of biomarkers of myocardial necrosis. Of these, we excluded the patients with age over 85 or hemodynamically unstable including mechanical support or in-hospital death, or others. Total 691 patients with hemodynamically stable and low clinical risk were analyzed. Detailed exclusion criteria are described in Fig 1. All patients were successfully underwent either emergent primary coronary intervention or emergent thrombolysis. Door-to-balloon time or symptom-onset-to-thrombolysis time was under 12 hours. If chest pain persisted over 12 hours, door-to-balloon time >12 hours or symptom-onset-to-thrombolysis time >12 hours could be included. All patients’ heart rhythm originated from the sinus node.

All patients received standard treatment including dual antiplatelet therapy, statins, beta-blockers, and angiotensin inhibitors as recommended for STEMI (Core measure of Korean Health Insurance Review & Assessment Service). Clinical outcomes were obtained from AMI registry (prospective observational study) and no patients were lost. The study protocol was approved by the Institutional Review board of Seoul National University Bundang Hospital and was conducted according to the Declaration of Helsinki. This research presents no more than minimal risk of harm to participants, and therefore IRB approved ‘Waiver of Documentation of Informed Consent’ (HRPP SOP II, IRB). All authors treated some portion of the patients in this study.

### Echocardiography

Echocardiography was performed within 24 hours after successful revascularization for all enrolled patients. Two-dimensional, M-mode, and Doppler echocardiography were performed in accordance with the American Society of Echocardiography guidelines. LV end-systolic and end-diastolic volumes along with the LVEF were calculated by the biplane Simpson’s method from apical 4- and 2-chamber views.

For global 2D strain analysis, a digital loop was acquired from parasternal short axis views at the apex, mid-papillary, and mitral valve level for circumferential strain, and from apical 4-chamber, 2-chamber, and apical long axis views for longitudinal strain. All images were transferred to NAS (Network-Attached Storage) and analyzed retrospectively. We traced along the LV endocardial border at the end-systolic frame. The strain curve was extracted from gray-scale images using dedicated software (EchoPac BT 12, GE Vingmed). Peak strain was defined as the peak negative value on the strain curve during the entire cardiac cycle. Peak GLS and GCS were calculated from the entire U-shaped (GLS) and circular-shaped (GCS) LV myocardium as: global strain (%) = (L[end-systole]–[end-diastole])/ L(end-diastole) × 100, where the global strain is the change of the whole myocardium, not an averaged value of each segmental strain, and L is the whole LV myocardium as one big segment. GLS was averaged from global strains from the apical 4-, 3- and 2-chamber views. GCS was averaged from 3 short axis (basal, mid and apical) views.

### Follow-up, outcome

All patients were seen within 4 weeks post-discharge and every 2 to 4 months thereafter for the duration of the follow-up period. The composite outcome included all-cause death, heart failure hospitalization, non-fatal myocardial infarction, and ventricular arrhythmia. Each factor was also analyzed individually.

### Statistical analysis

All data are expressed as mean value ± standard deviation, if not otherwise specified. Outcome data were compared between subjects by Cox-regression model. P-values less than 0.05 were considered to indicate statistically significant differences. To minimize correlation effects, we used 5 Cox proportional hazards models, and analyzed LVEF, GCS, and GLS separately. Model 1 only adjusted for age. Model 2 included adjustments for age, hypertension, DES (drug eluting stent) implantation, and WMSI (wall motion score index). Model 3 adjustments included factors in model 2 and LVEF, and Model 4 adjusted factors in model 2 and GLS. Model 5 included adjusted factors in model 2 and GCS. The incremental prognostic value was defined by a significant increase in global chi-square.

Receiver-operating characteristic (ROC) curve analysis was used to determine optimal cutoff values of continuous variables. The best cutoff value was defined as the point with the highest sum of sensitivity and specificity. The subjects were divided into three groups according to their LVEF as preserved (EF > 50%), mild depressed (40–50%), and significantly depressed (EF < 40%).

All analyses were performed with IBM SPSS Statistics Version 21.0 (IBM/SPSS, Chicago, IL).

### Feasibility and reproducibility

Of total 691 patients, and global strain was acquired in 678 (98.1%) patients for longitudinal strain and in 653 (94.5%) patients for circumferential strain. Variability in the measurement of strain was evaluated in 20 randomly selected patients. For intra-observer variability, the same observer measured strain for each of the selected patients again 15 days later. The interclass correlation coefficiency of intra-observer variability for GLS and GCS were 0.96 and 0.94 respectively. For the inter-observer variability, a second independent observer repeated the analysis. The interclass correlation coefficiency of inter-observer variability for GLS and GCS were 0.94 and 0.88 respectively.

## Results

### Baseline characteristics

Total 691 consecutive patients (mean age 59.3 ± 13.1) satisfied all inclusion criteria. During mean follow-up duration of 39 ± 19 months, clinical events occurred in 47 patients (6.8%), including 19 deaths (12 cardiac deaths), 14 heart failure hospitalizations, 13 myocardial infarctions, and 1 non-fatal ventricular arrhythmia. The clinical and echocardiographic characteristics are described in Table 1. Compared with patients who did not develop clinical events, patients with events were older, had more medical history of hypertension and DES implantation, had a greater left atrial volume and LV end-systolic diameter, LV mass, had lower LVEF, and had higher global GLS and GCS.

**Table 1:** 

	Total (N = 691)	Composite outcome		P Value
		No (N = 644)	Yes (N = 47)	
Age	59.3 ± 13.2	58.7 ± 13.1	67.5 ± 11.4	**<0.001**
Female	139 (20.1%)	125 (19.4%)	14 (19.4%)	0.068
Weight (kg)	67.8 ± 11.5	68.1 ± 11.4	63.6 ± 11.1	0.010
Height (cm)	165.9 ± 8.2	166.2 ± 8.1	163.1 ± 8.2	**0.018**
Hypertension	323 (46.7%)	293 (45.5%)	30 (63.8%)	**0.011**
Diabetes	162 (23.4%)	149 (23.1%)	13 (27.7%)	0.292
FHx. of CAD	70 (10.1%)	68 (10.6%)	2 (4.3%)	0.123
Previous PCI or CABG	25 (3.6%)	22 (3.4%)	3 (6.4%)	0.238
Culprit LM	1 (0.1%)	1 (0.2%)	0 (0.0%)	0.932
LAD	368 (53.3%)	337 (52.3%)	31 (66.0%)	**0.048**
LCX	64 (9.3%)	60 (9.3%)	4 (8.5%)	0.555
RCA	233 (33.7%)	224 (34.8%)	9 (19.1%)	0.018
Treatment: PCI	647 (93.6%)	603 (93.6%)	44 (93.6%)	0.590
Thrombolysis	44 (6.4%)	41 (6.4%)	3 (6.4%)	
DES implantation	507 (78.5%)	507 (79.0%)	31 (66.0%)	**0.033**
Killip class	1.1 ± 0.6	1.1 ± 0.5	1.0 ± 0.7.	0.129
0	32 (4.6%)	22 (3.4%)	10 (21.3%)	**<0.001**
1	566 (81.9%)	536 (83.2%)	30 (63.8%)	**0.002**
2	71 (10.3%)	66 (10.2%)	5 (10.6%)	0.542
3	15 (2.2%)	13 (2.0%)	2 (4.3%)	0.272
4	7 (1.0%)	7 (1.1%)	0 (0.0%)	0.609
Creatinine	1.0 ± 0.7	1.0 ± 0.7	1.0 ± 0.3	0.610
Total cholesterol	198.3 ± 48.0	198.0 ± 42.2	201.1 ± 44.1	0.654
LDL cholesterol	120.0 ± 40.0	120.6 ± 39.5	111.9 ± 45.3	0.238
LV mass Index, g/m^2^	107.6 ± 26.5	106.7 ± 25.7	120.0 ± 39.5	**0.001**
LV dimension, mm				
End-systole	34.1 ± 6.0	33.9 ± 5.9	37.6 ± 6.8	**0.001**
End-diastole	49.3 ± 17.3	49.2 ± 17.9	50.2 ± 5.5	0.379
LV volume, ml				
End-systole	43.1 ± 17.1	42.4 ± 16.2	52.4 ± 25.6	**<0.001**
End-diastole	85.6 ± 22.9	85.4 ± 22.5	88.4 ± 27.9	0.382
LV ejection fraction, %	50.8 ± 9.8	51.3 ± 9.3	43.4 ± 13.7	**<0.001**
WMSI	1.58 ± 0.3	1.57 ± 0.3	1.72 ± 0.5	0.078
LA volume index, ml/m2	30.4 ± 9.8	29.9 ± 9.2	37.3 ± 14.2	**<0.001**
Global GLS, %	–13.0 ± 3.7	–13.2 ± 3.6	–10.1 ± 4.0	**<0.001**
Global GCS, %	–15.3 ± 4.9	–15.5 ± 4.8	–13.2 ± 5.5	**0.012**
Follow-up duration (months)	38.6 ± 19.2	39.6 ± 18.7	25.3 ± 20.7	<0.001

FHx of CAD, Family history of Coronary Artery Disease; PCI, Percutaneous Coronary Intervention; CABG, Coronary Artery Bypass Graft surgery; LM. left main coronary artery; LAD, left anterior descending coronary artery; LCX, left circumflex artery; RCA, Right coronary artery; DES, drug-eluting stent; LDL, low-density lipoprotein cholesterol; LV, left ventricle; WMSI, wall motion score index; LA, left atrium; GLS, global longitudinal strain; GCS, global circumferential strain

### Predictors of composite outcome

By univariate analysis using Cox proportional hazards model, age, hypertension, DES implantation, WMSI, LVESD (left ventricular end systolic diameter), LVEF, and both GLS and GCS were significantly associated with composite outcome. The hazard ratio (HR) of each variable is shown in Table 2. Among the nine variables considered to be related to composite outcome, only two (age and GLS) were relevant in the multivariate Cox analysis, and GLS was the strongest independent predictor of composite outcome. Using 5 survival models described in the methods section, GLS was the strongest independent predictor of composite outcome, regardless of adjusted variables (S1 Table). Moreover, GLS offers an incremental value over conventional LVEF (χ2 = 31.8→45.8, p<0.001), while GCS offers no additional benefits.

**Table 2:** 

	Univariate Analysis			Multivariate Analysis*		
Variable	HR	95% CI	P Value	HR	95% CI	P Value
Age	1.06	1.03–1.08	**<0.001**	**1.08**	**1.04–1.13**	**0.001**
Female	0.78	0.95–3.32	0.072			
Hypertension	2.07	1.14–3.76	**0.016**	0.94	0.39–2.24	0.891
Diabetes	1.28	0.68–2.43	0.448			
LAD culprit	1.71	0.94–3.12	0.082			
PCI	0.96	0.30–3.10	0.948			
DES	0.53	0.29–0.97	**0.039**	1.17	0.46–2.94	0.745
Killip class >1	1.27	0.57–2.85	0.556			
LVESD	1.09	1.05–1.13	**<0.001**	1.06	0.98–1.05	0.693
LVEDD	1.00	0.99–1.01	0.806			
LAVI	1.06	1.03–1.08	**<0.001**	1.01	0.97–1.05	0.693
LVEF	1.08	0.05–1.10	**<0.001**	1.05	0.99–1.12	0.120
GLS (%)	1.33	1.21–1.46	**<0.001**	**1.37**	**1.13–1.66**	**0.001**
GCS (%)	1.11	1.04–1.20	**0.004**	0.94	0.82–1.07	0.327

* Adjusted with age, hypertension, DES, WMSI, LVESD, LAVI, LVEF, GLS and GCS.

**Fig 2:**
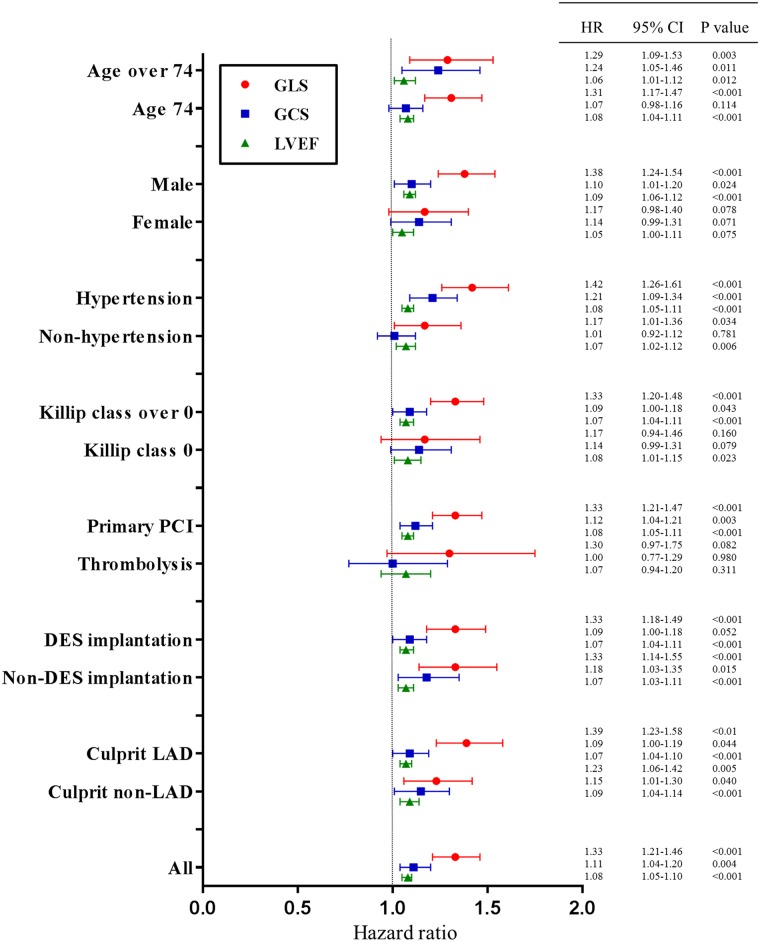
The prognostic power of 2D-strain and LVEF in the subgroup analysis. To exclude the confounding effects of clinical risk factors effect, the subgroup analysis of each factor were performed. The risk factors include age≥75 (n = 104), male (n = 552), hypertension (n = 323), Killip class >0 (n = 659), primary PCI (n = 647), DES implantation (n = 507), and culprit of LAD (n = 368).

To exclude confounding effects of clinical risk factors, subgroup analyses of each factor were performed. Detailed data according to each factor is described in Fig 2. GLS showed significant predictive power in most subgroups except female, patients with thrombolysis, and low risk patients with Killip class 0.

By analyzing the ROC curve, the best cut-off points of GLS (AUC 0.74, 95% CI 0.65–0.83, p<0.001) and GCS (AUC 0.63, 95% CI 0.53–0.72, p = 0.005) were -9.9% (sensitivity 65%, specificity 84%) and -12.6% (sensitivity 54%, specificity 73%), respectively. Patients with GLS>-9.9% and GCS>-12.6% showed 8.0 and 3.0 times higher risk of composite events than patients with GLS ≤9.9% and GSC ≤12.6%, respectively.

### Predictive power of GLS and GCS according to LVEF

**Fig 3:**
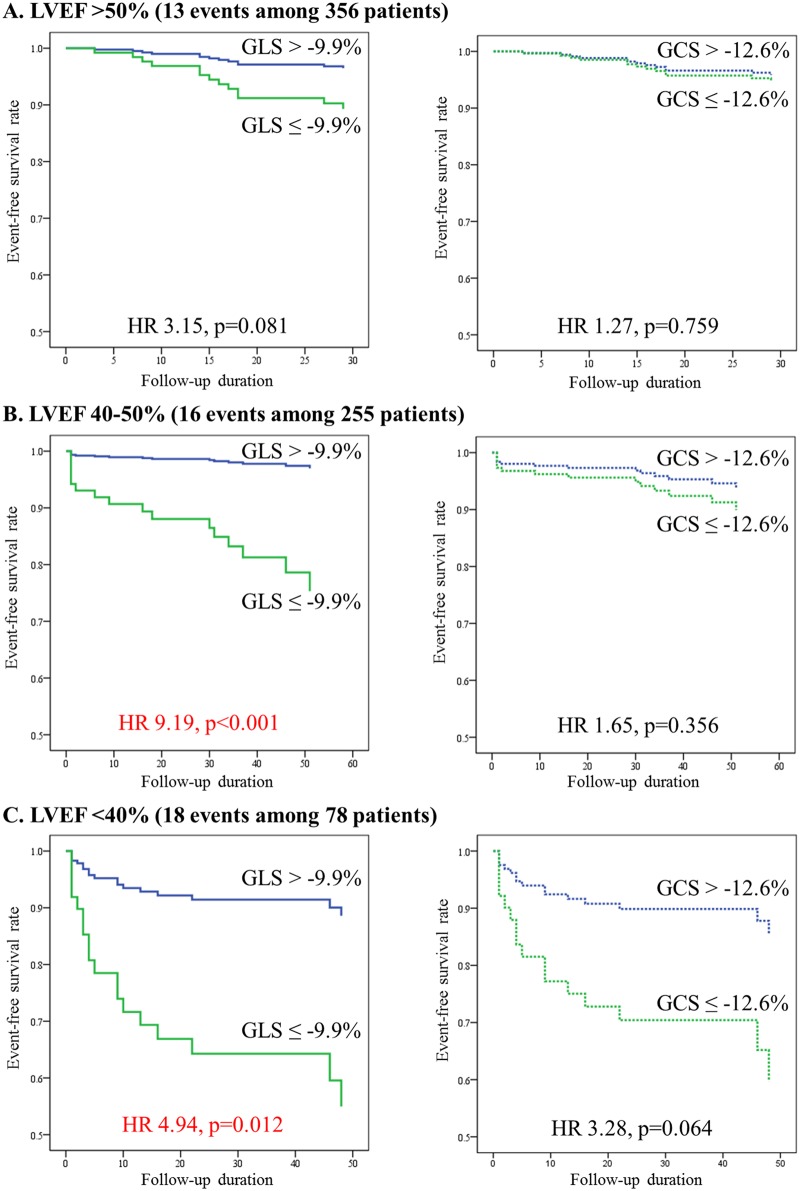
Kaplan-Meier plots of GLS according to LVEF. Kaplan-Meier plots according to LVEF of preserved (EF > 50%), mild depressed (40–50%), and significantly depressed (< 40%) systolic function. A) EF>50% (13 events among 356 patients). B) LVEF 40–50% (16 events among 255 patients). C) LVEF<40% (18 events among 78 patients). GLS is a powerful predictor of clinical events and a better parameter than GCS in successfully reperfused STEMI patients, especially with significant LV dysfunction. Global strains did not predict outcome for the patients with preserved LVEF.

Patients were categorized into three groups according to LVEF (preserved / mildly depressed / significantly depressed). In survival analysis according to LVEF, GCS lost its predictive power. GLS was the most powerful independent predictor for patients with mildly and significantly depressed LVEF, but lost its significance in the preserved LVEF group (Fig 3). Among patient with LVEF <40%, GLS>-9.9% patients showed 5 times higher risk than those with GLS<-9.9%. By survival analyses using model 2–3 to adjust for clinical risk factors and LVEF, both GLS and GCS could not predict outcomes for patients with preserved LV function. For the patients with significantly depressed LV systolic function, GLS still showed the best predictive power among three factors. However GCS and LVEF lost their significance for event prediction with significantly depressed LV function (<40%). The detailed data is described in (Table 3).

**Table 3:** 

**LVEF >50%**	Number of events per total patients	Model 2, HR (95% CI)	Model 3, HR (95% CI)
GLS	13/356	1.03 (0.81–1.32)	0.96 (0.83–1.11)
p-value		0.800	0.614
GCS		1.00 (0.85–1.17)	0.96 (0.83–1.11)
p-value		0.975	0.572
LVEF		0.96 (0.83–1.11)	-
p-value		0.617	-
**LVEF 40–50%**		Model 2	Model 3
GLS	16/255	**2.25 (1.44–3.50)**	**2.47 (1.47–4.15)**
p-value		**<0.001**	**0.001**
GCS		**1.55 (1.09–2.21)**	**1.54 (1.08–2.20)**
p-value		**0.014**	**0.018**
LVEF		**1.42 (1.11–1.82)**	**-**
p-value		**0.006**	**-**
**LVEF <40%**		Model 2	Model 3
GLS	18/78	**1.28 (1.05–1.56)**	**1.30 (1.07–1.58)**
p-value		**0.016**	**0.010**
GCS		1.14 (0.99–1.31)	1.15 (1.00–1.32)
p-value		0.070	0.059
LVEF		1.08 (0.98–1.18)	-
p-value		0.116	-

Model 2 included adjustment for age and hypertension, DES, WMSI. Model 3 included factors in model 2 and LVEF

### Individual components of composite events analysis

There were 19 (2.7%) deaths (including 12 cardiac deaths), 14 (2.0%) hospitalizations for heart failure, 13 (1.9%) cases of myocardial infarction, and one (0.1%) non-fatal ventricular arrhythmia. The GLS, GCS, and LVEF mean values for composite outcomes and each individual components are described in S2 Table. GLS, GCS, and LVEF did not differ in regard to the occurrence of myocardial infarction.

**Fig 4:**
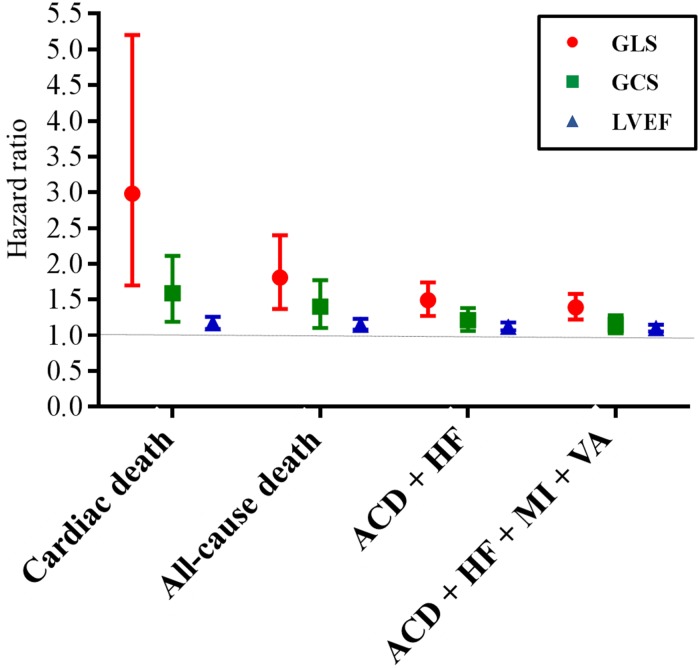
The composite outcome and its individual components, adjusted with clinical factors. ACD = all-cause death, HF = heart failure, MI = Myocardial infarction, VA = non-fatal ventricular arrhythmia. Although GLS, GCS, and LVEF were predictors for composite outcome, their prediction power was best for cardiac death.

Cox-regression survival analysis for composite outcomes and each individual components, using model 2, showed GLS to be the most powerful predictive power, rather than GCS or LVEF. Although GLS, GCS, and LVEF were predictors for composite outcome, their prediction power was best for cardiac death (Fig 4). Detailed data are presented in S3 Table.

## Discussion

The role of global 2D-strain in AMI patients engaged clinicians’ interest and a number of researches were reported recently.[13–15] Global strain has been introduced and validated using tagged magnetic resonance imaging and sonomicrometry.[16–18] Recent study reported that longitudinal and circumferential strains correlate with myocardial infarct mass and significantly differentiate the size of myocardial infarcts.[19] Lots of echocardiographic parameters have been introduced during the last decades for assessment of myocardial function in AMI patients. GLS has been discussed in numerous studies regarding acute myocardial infarction, and the association between GCS and the prognosis of heart failure patients is well known. We hypothesized that both global longitudinal and circumferential strain might be reliable and feasible clinical tools for risk stratification and could substitute LVEF in STEMI patients with both preserved and reduced LV systolic function.

We have shown that GLS and was the best independent prognostic factors to predict composite events in successfully revascularized STEMI patients. GLS showed incremental predictive effect in addition to LVEF. Subgroup analysis according to baseline LV systolic function, for the patients with significantly depressed LV systolic function (<40%), GLS showed the powerful predictive power, although GCS and LVEF lost predictive power.

After successfully reperfused therapy in STEMI, dysfunctional myocardial segments subtended by the infarct-related artery can follow two different natural courses: functional recovery or irreversible remodeling.[20] Irreversible remodeling of LV myocardium would be linked to poorer outcome, and LV remodeling after AMI is an important precursor of the development of overt heart failure and is an important predictor of mortality.[21] GLS showed excellent prediction of LV remodeling and adverse clinical events in patients with anterior wall AMI.[5] In the current study, we validated the clinical usefulness of measurement of global strain addition to LVEF, and also showed that global strain had an important advantage when LVEF was low to predict outcome. This is very important because many STEMI patients have low LVEF immediately after revascularization. In our data, in patients with significant LV dysfunction (LVEF < 40%), LVEF could not have predictive power, although GLS showed powerful predictive power. We thought that the reason for poor prediction of LVEF might be due to the low accuracy of LVEF in patients with significant LV dysfunction. The LVEF can often be overestimated and reproducibility of LVEF has known to be low, especially for those with distorted LV geometry and/or functional mitral regurgitation with LV dysfunction. Both LVEF and strain are sensitive to myocardial contractility, although they are known to be both load-dependent. However global strain is less dependent on afterload than LVEF for the failing heart,[22] therefore global strain can reflect contractility more accurately than LVEF in a situation of exposure to acute pressure change such as acute myocardial infarction. In previous studies, global strain was better indicator for LV contractile function than LVEF.[23] In previous study from JK Oh et al., the investigators re-measured (by the Core Lab) the LVEF data from STITCH Trial [24] which included patients with LVEF under 35%. The result showed that 18.5% of patients in STITCH Trial were found to have LVEF greater than 35%.[25] Of the total patients, 10.6% had 35 < LVEF ≤40% and 7.9% had LVEF >40%. It is well known that global strain showed better reproducibility and feasibility than LVEF.[26] The authors say that global strain is better parameter in various respect than LVEF especially for failing heart.

The GLS did not predict outcome for the patients with preserved LVEF (LVEF >50%). In the present study the most important reason LVEF lost its predictive power in the group with preserved LVEF was, that there were only 13 (3.6%) clinical events among 356 patients, which was too small a number to have statistical power. when we set the cut-off value of preserved LVEF as 40%, GLS showed significant predictive power as previously reported.[4]

Contraction of muscle fibers in the mid-wall, which is linearly related to circumferential strain,[8] may better reflect intrinsic contractility than contraction of fibers in the endocardium.[9] However, there are few data on the use of circumferential strain as a predictor in AMI patients. A recent paper reported that both longitudinal and circumferential strain rates were independent predictors of outcomes after MI, whereas only circumferential strain rates, but not longitudinal strain rates were predictive of remodeling, suggesting that preserved circumferential function might serve.[27] Preserved midwall contraction assessed by circumferential strain is also very important parameter in heart failure.[10] In our data, however, the predictive power of GLS was superior to that of GCS. We assume that in STEMI patient, the main component of ischemia vulnerable myocardium in AMI is subendocardial layer, which is reflected to GLS.

Many factors influence the prognosis of patients with STEMI. We performed further analyses on cardiac biomarker, intraventricular conduction delay (QRS duration), and the history of previous PCI or CABG. Infarct size has been known to be a valuable surrogate marker for patients with myocardial infarction. However a study which has investigated predictors of 6-month cardiac events after myocardial infarction from the Korea Acute Myocardial Infarction Registry (KAMIR) showed that cardiac biomarkers did not predict clinical outcome.[28] In our study cohort, The peak CK-MB level could not predict clinical outcome and had no additional predictive power to LVEF and global strains when we adjusted for CK-MB as a confounding factor in Cox-regression analyses (S4 Table). There were 50 (7.2%) patients whose QRS duration was over 120 msec. There was no significant difference of mean QRS duration between patients with or without clinical events (99.7 ± 22.1 versus 96.0 ± 23.5, *p* = 0.266). In the univariate and multivariate analyses for study outcome, QRS duration could not predict clinical outcome and the predictive power of LVEF and global strains did not change although we corrected QRS duration as confounding in Cox-regression analyses (S5 Table). Also, we re-analyzed the predictive power of GLS, GCS and LVEF after excluding patients with history of previous PCI or CABG (S6 Table)). There was no change in the conclusion after the re-analysis.

A number of limitations of this analysis should be noted. Cardiac death was significantly lower than previous published data. Although this was a prospective analysis performed at multi-center, lots of patients (n = 107) with mechanical support, ongoing cardiogenic shock, age over 85 or cardiac arrest at admission, were excluded from the analysis. It could be a baseline selection bias. This meant the patients who could be thought to have poorer outcome were already excluded before analysis. The patients in our study are those who would benefit most from the right treatment strategy according to the outcome prediction. Following strict inclusion criteria which could lead a selection bias, we were able to control various confounding factors that could influence prognosis. All study patients were stable enough to perform routine echocardiography at a dedicated echocardiogram exam center. Even so, 1 patient had a poor echocardiography window, and was excluded from the study. LVEF was acquired in all 691 patients, and global strain was acquired in 678 (98.1%) patients. Secondly, we could not undergo the analysis of scheduled follow-up echocardiography, we could not definitely address 2D-strain is associate with LV remodeling in our cohort. Finally, vagaries of coronary anatomy, morphology, combined non-culprit stenosis, or TIMI score for STEMI patients are important factors in the aspect of clinical prognosis. We did not reflect these interventional data on our study, and it might affect the results as confounding bias.

## Conclusion

GLS is a powerful predictor of clinical events and appears to be a better parameter than ejection fraction in successfully reperfused STEMI patients, especially with LV dysfunction (LVEF ≤50%), although GCS and LVEF lost their predictive power for the patients with significantly depressed LV function (LVEF <40%) These data further support the concept that longitudinal function is sensitive to acute myocardial damage in STEMI patients. However GLS did not predict outcome for the patients with preserved LVEF (LVEF >50%).

## Supporting information

S1 Table Adjusted hazard ratio for composite outcome (death, heart failure hospitalization, myocardial infarction, and ventricular arrhythmia) using five models. (DOCX)

S2 Table The GLS, GCS and LVEF mean values of each individual components and outcome A to D. (DOCX)

S3 Table The cox-regression analysis of the composite outcome and its individual components, adjusted with clinical factors. (DOCX)

S4 Table The cox-regression analysis of the composite outcome and cardiac biomarker (peak CK-MB), adjusted with clinical factors. (DOCX)

S5 Table The cox-regression analysis of the composite outcome and intraventricular conduction delay (QRS duration), adjusted with clinical factors. (DOCX)

S6 Table The cox-regression analysis of the composite outcome after excluding patients with PCI or CABG history, adjusted with clinical factors. (DOCX)
