# Correction: Prognostic power of global 2D strain according to left ventricular ejection fraction in patients with ST elevation myocardial infarction

**DOI:** 10.1371/journal.pone.0186437

**Published:** 2017-10-10

**Authors:** Myung-Jin Cha, Hyun-Sook Kim, Seong Hwan Kim, Jae-Hyeong Park, Goo-Yeong Cho

There is an error in affiliation 5 for author Goo-Yeong Cho. Affiliation 5 should be: Division of Cardiology, Cardiovascular Center, College of Medicine, Seoul National University, Seoul National University Bundang Hospital, Seongnam, South Korea.
