# Supplementary material for: Prognostic power of global 2D strain according to left ventricular ejection fraction in patients with ST elevation myocardial infarction
Source: PLoS One. 2017 Mar 23;12(3):e0174160. doi: 10.1371/journal.pone.0174160 (PMC5363861; doi:10.1371/journal.pone.0174160)
Supplement: S1 Table — (DOCX) [file pone.0174160.s001.docx]

**S1 Table. Adjusted Hazard Ratio for composite outcome (death, heart failure hospitalization, myocardial infarction, and ventricular arrhythmia) using five models.**

| **LVEF** | Model 1 | Model 2 | Model 3 | Model 4 | Model 5 |
| --- | --- | --- | --- | --- | --- |
| HR  (95% CI) | **1.07**  **0.04-1.10** | **1.10**  **1.05-1.15** | -  - | **1.07**  **1.03-1.12** | **1.09**  **1.04-1.15** |
| p-value | **<0.001** | **<0.001** | - | **0.002** | **<0.001** |
| **GLS** | Model 1 | Model 2 | Model 3 | Model 4 | Model 5 |
| HR  (95% CI) | **1.29**  **1.17-1.42** | **1.39**  **1.22-1.58** | **1.30**  **1.15-1.48** | **-**  **-** | **1.38**  **1.16-1.63** |
| p-value | **<0.001** | **<0.001** | **<0.001** | **-** | **<0.001** |
| **GCS** | Model 1 | Model 2 | Model 3 | Model 4 | Model 5 |
| HR  (95% CI) | **1.10**  **1.03-1.19** | **1.15**  **1.03-1.28** | **1.11**  **1.00-1.26** | 0.99  0.88-1.11 | -  - |
| p-value | **0.008** | **0.011** | **0.048** | 0.868 | - |

Model 1 adjusted only age factor. Model 2 included adjustment for age and hypertension, DES, WMSI. Model 3 included factors in model 2 and LVEF, and Model 4 adjusted factors in model 2 and GLS. Model 5 included adjusted factors in model 2 and GCS
