# Supplementary material for: Prognostic power of global 2D strain according to left ventricular ejection fraction in patients with ST elevation myocardial infarction
Source: PLoS One. 2017 Mar 23;12(3):e0174160. doi: 10.1371/journal.pone.0174160 (PMC5363861; doi:10.1371/journal.pone.0174160)
Supplement: S2 Table — (DOCX) [file pone.0174160.s002.docx]

**S2 Table. The GLS, GCS and LVEF mean values of each individual components and outcome A to D.**

|  |  | **GLS** | **GCS** | **LVEF** |
| --- | --- | --- | --- | --- |
| **Cardiac death** | yes | 7.2 ± 1.9 | 9.5 ± 3.6 | 35.7 ± 7.9 |
| **(n=12)** | no | 13.1 ± 3.6 | 15.4 ± 4.9 | 51.0 ± 9.6 |
|  | *p* | ***<0.001*** | ***<0.001*** | ***<0.001*** |
| **All-cause death** | yes | 9.2 ± 4.0 | 11.2 ± 4.4 | 40.4 ± 12.2 |
| **(n=19)** | no | 13.1 ± 3.6 | 15.5 ± 4.9 | 51.1 ± 9.6 |
|  | *p* | ***0.001*** | ***0.001*** | ***0.001*** |
| **HF hospitalization** | yes | 9.5 ± 3.5 | 12.6 ± 4.9 | 38.8 ± 12.1 |
| **(n=14)** | no | 13.0 ± 3.7 | 15.4 ± 4.8 | 51.0 ± 9.6 |
|  | *p* | ***0.003*** | *0.070* | ***0.002*** |
| **Myocardial infarction** | yes | 12.0 ± 4.2 | 16.4 ± 6.4 | 53.7 ± 13.1 |
| **(n=13)** | no | 13.0 ± 3.7 | 15.3 ± 4.9 | 50.7 ± 9.8 |
|  | *p* | *0.406* | *0.559* | *0.422* |
| **Non-fatal ventricular arrhythmia** | yes | 11.4 | 14.8 | 31.8 |
| **(n=1)** | no | 13.0 ± 3.7 | 15.3 ± 4.9 | 50.8 ± 9.8 |
|  | *p* | *-* | *-* | *-* |
| **ACD + HF** | yes | 9.4 ± 3.7 | 11.8 ± 4.6 | 39.7 ± 12.0 |
| **(n=26, 3.8%)** | no | 13.2 ± 3.6 | 15.5 ± 4.8 | 51.3 ± 9.4 |
|  | *p* | ***<0.001*** | ***<0.001*** | ***<0.001*** |
| **ACD + HF + MI + VA** | yes | 10.1 ± 4.0 | 13.2 ± 5.5 | 13.4 ± 13.7 |
| **(n=56, 8.1%)** | no | 13.2 ± 3.6 | 15.5 ± 4.8 | 51.3 ± 9.3 |
|  | *p* | ***<0.001*** | ***0.012*** | ***<0.001*** |

ACD=all-cause death, HF=heart failure, MI=Myocardial infarction, VA=non-fatal ventricular arrhythmia
