# Supplementary material for: Prognostic power of global 2D strain according to left ventricular ejection fraction in patients with ST elevation myocardial infarction
Source: PLoS One. 2017 Mar 23;12(3):e0174160. doi: 10.1371/journal.pone.0174160 (PMC5363861; doi:10.1371/journal.pone.0174160)
Supplement: S3 Table — (DOCX) [file pone.0174160.s003.docx]

**S3 Table. The cox-regression analysis of the composite outcome and its individual components, adjusted with clinical factors**

|  | ***GLS** | | |  | ***GCS** | | |  | ***LVEF** | | |
| --- | --- | --- | --- | --- | --- | --- | --- | --- | --- | --- | --- |
|  | HR | 95% CI | p-value |  | HR | 95% CI | p-value |  | HR | 95% CI | p-value |
| Cardiac death | 2.98 | 1.70-5.20 | **<0.001** |  | 1.59 | 1.19-2.11 | **0.001** |  | 1.17 | 1.09-1.26 | **<0.001** |
| All-cause death | 1.81 | 1.37-2.40 | **<0.001** |  | 1.40 | 1.10-1.77 | **0.005** |  | 1.15 | 1.08-1.23 | **<0.001** |
| ACD + HF | 1.49 | 1.27-1.74 | **<0.001** |  | 1.21 | 1.06-1.38 | **0.006** |  | 1.12 | 1.07-1.18 | **<0.001** |
| ACD + HF + MI + VA | 1.39 | 1.22-1.58 | **<0.001** |  | 1.15 | 1.03-1.28 | **0.011** |  | 1.10 | 1.05-1.15 | **<0.001** |

* Adjusted with age and hypertension, DES, WMSI

ACD=all-cause death, HF=heart failure, MI=Myocardial infarction, VA=non-fatal ventricular arrhythmia
