# Supplementary material for: Prognostic power of global 2D strain according to left ventricular ejection fraction in patients with ST elevation myocardial infarction
Source: PLoS One. 2017 Mar 23;12(3):e0174160. doi: 10.1371/journal.pone.0174160 (PMC5363861; doi:10.1371/journal.pone.0174160)
Supplement: S5 Table — (DOCX) [file pone.0174160.s005.docx]

**S5 Table. The cox-regression analysis of the composite outcome and intraventricular conduction delay (QRS duration), adjusted with clinical factors**

|  | Univariate Analysis | | | Multivariate Analysis* | | |
| --- | --- | --- | --- | --- | --- | --- |
| Variable | HR | 95% CI | P Value | HR | 95% CI | P Value |
| QRS duration | 1.00 | 0.99-1.01 | 0.324 | 1.00 | 0.99-1.02 | 0.902 |
| LVEF | 1.08 | 0.05-1.10 | **<0.001** | 1.05 | 0.99-1.12 | 0.136 |
| GLS (%) | 1.33 | 1.21-1.46 | **<0.001** | **1.37** | **1.13-1.66** | **0.001** |
| GCS (%) | 1.11 | 1.04-1.20 | **0.004** | 0.94 | 0.82-1.08 | 0.350 |

* Adjusted with age, hypertension, DES, WMSI, LVESD, LAVI
