# Supplementary material for: Prognostic power of global 2D strain according to left ventricular ejection fraction in patients with ST elevation myocardial infarction
Source: PLoS One. 2017 Mar 23;12(3):e0174160. doi: 10.1371/journal.pone.0174160 (PMC5363861; doi:10.1371/journal.pone.0174160)
Supplement: S6 Table — (DOCX) [file pone.0174160.s006.docx]

**S6 Table. The cox-regression analysis of the composite outcome after excluding patients with PCI or CABG history, adjusted with clinical factors**

|  | Univariate Analysis | | | Multivariate Analysis* | | |
| --- | --- | --- | --- | --- | --- | --- |
| Variable | HR | 95% CI | P Value | HR | 95% CI | P Value |
| LVEF | 1.08 | 1.05-1.11 | <0.001 | 1.04 | 0.98-1.12 | 0.196 |
| GLS (%) | **1.34** | **1.22-1.48** | **<0.001** | **1.37** | **1.12-1.67** | **0.002** |
| GCS (%) | 1.12 | 1.04-1.21 | 0.004 | 0.96 | 0.83-1.11 | 0.577 |
